# Hypoxia Mimetic Agents for Ischemic Stroke

**DOI:** 10.3389/fcell.2018.00175

**Published:** 2019-01-08

**Authors:** Charles K. Davis, Saurabh A. Jain, Ok-Nam Bae, Arshad Majid, G. K. Rajanikant

**Affiliations:** ^1^School of Biotechnology, National Institute of Technology Calicut, Calicut, India; ^2^Sheffield Institute for Translational Neuroscience, University of Sheffield, Sheffield, United Kingdom; ^3^College of Pharmacy, Hanyang University, Ansan, South Korea

**Keywords:** hypoxia-inducible factor-1, ischemic stroke, neuroprotection, iron chelators, hypoxia mimetic agent

## Abstract

Every year stroke claims more than 6 million lives worldwide. The majority of them are ischemic stroke. Small molecule-based therapeutics for ischemic stroke has attracted a lot of attention, but none has been shown to be clinically useful so far. Hypoxia-inducible factor-1 (HIF-1) plays a crucial role in the transcriptional adaptation of cells to hypoxia. Small molecule-based hypoxia-mimetic agents either stabilize HIF-1α *via* HIF-prolyl hydroxylases (PHDs) inhibition or through other mechanisms. In both the cases, these agents have been shown to confer ischemic neuroprotection *in vitro* and *in vivo*. The agents which act *via* PHD inhibition are mainly classified into iron chelators, iron competitors, and 2 oxoglutarate (2OG) analogs. This review discusses HIF structure and key players in the HIF-1 degradation pathway as well as the genes, proteins and chemical molecules that are connected to HIF-1 and how they affect cell survival following ischemic injury. Furthermore, this review gives a summary of studies that used PHD inhibitors and other HIF-1α stabilizers as hypoxia-mimetic agents for the treatment of ischemic injury.

## Introduction

**FIGURE 1:**
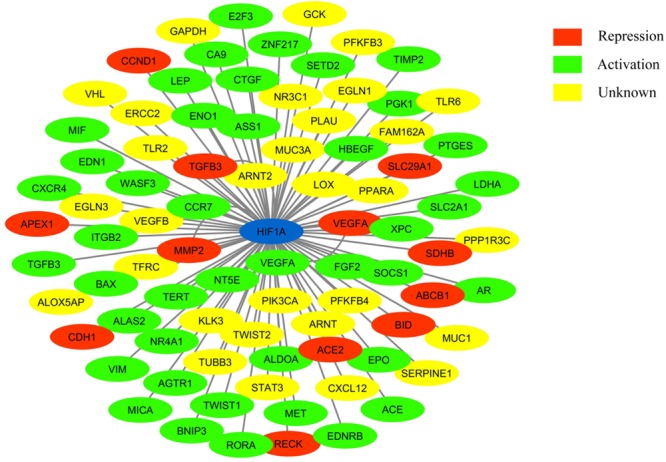
Network showing HIF-1 connected to its target genes (adapted from: http://www.grnpedia.org/trrust/). Colors inside the shape represent the type of interaction.

HIF-1 is a transcription factor that acts as a master regulator of O_2_ homeostasis (Semenza, 1998). It modulates the expression of an array of genes (Figure 1), and many of them are involved in the cellular adaptation to hypoxia (Semenza, 2001). Vascular endothelial growth factor (VEGF), Erythropoietin (EPO), glucose transporter 1 (GLUT1), heme oxygenase 1 (HO-1), and endothelial nitric oxide (eNOS) synthase are some of the genes that are directly involved in HIF-1 mediated cellular response to hypoxia (Schofield and Ratcliffe, 2004). As HIF-1 stabilization is an endogenous cellular mechanism to reduce hypoxia-induced injury, it is hypothesized that pre and post-hypoxic stabilization of HIF-1 by external means can enhance protection (Shi, 2009). Among these foreign interventions, the small molecule-based HIF-1 stabilization is widely studied and has shown promising results (Siddiq et al., 2005). Most of the small-molecules used for this purpose either mimic or compete with the co-factors or co-substrates of HIF-1 degradation pathway. The small molecules that are used to stabilize HIF-1α are widely known as hypoxia-mimetic agents. Some of the familiar hypoxia-mimetic agents are desferrioxamine (DFO) and cobalt chloride (CoCl_2_) (Zeng et al., 2011). Apart from these small molecules, there are newly found mRNAs and other compounds which stabilize HIF-1α. In this review, we discuss the small chemical molecules and other biological compounds that have conferred protection against ischemic stroke injury *via* HIF-1 pathway stabilization.

## HIF Structure and Pathway

**FIGURE 2:**
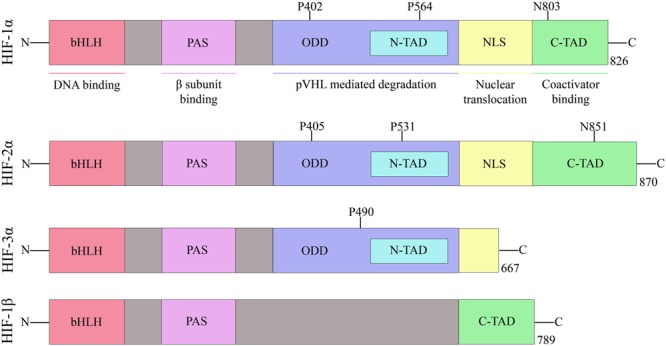
Domain structure of human HIF subunits and isoforms.

Hypoxia-inducible factor belongs to the PAS family (PER-ARNT-SIM), and functionally active HIF consists of two basic helix-loop-helix (bHLH) protein subunits, alpha and beta/ARNT (aryl hydrocarbon receptor nuclear translocator). Apart from bHLH, N-terminal half of HIFs composed of Per-ARNT-Sim (PAS) homology domains. The bHLH and PAS regions are involved in the heterodimerization and DNA binding, respectively. The terminal-transactivation domains (TADs) are present in the C-terminal region of HIF, and they control the transcriptional activity of the protein (Figure 2) (Beaudry et al., 2016).

**FIGURE 3:**
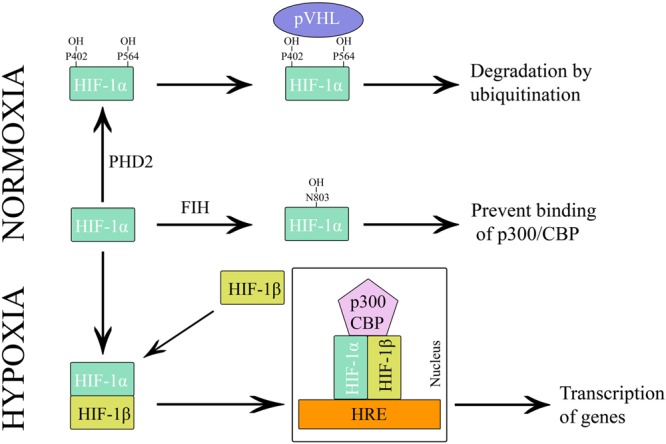
Regulation of HIF-1 during hypoxia and normoxia.

The alpha subunit of HIF has three isoforms, HIF-1α, HIF-2α, and HIF-3α. Likewise, the beta subunit has three isoforms, ARNT, ARNT2, and ARNT3 (Semenza, 2000). The HIF-1α/β dimer controls the transcription of genes by binding to a core DNA motif (G/ACGTG) in hypoxia-response elements (HREs). The HIF-1β subunit is constitutively produced in the nucleus whereas the stability of HIF-1α depends on the availability of oxygen (Vadlapatla et al., 2013). Under hypoxic condition, HIF-1α is stable and transported to the nucleus. However, under the normoxic condition, HIF mediated transcription is inhibited by the HIF prolyl hydroxylases (PHDs) and asparaginyl hydroxylase (FIH). PHD2 hydroxylates the two proline residues (402 and 564) on the oxygen-dependent degradation domain (ODDD) of HIF-1α and makes it susceptible to ubiquitin-mediated proteolysis *via* von Hippel–Lindau tumour suppressor (pVHL) binding. On the contrary, FIH hydroxylases asparagine residue (803) of HIF-1α and prevents binding of the transcriptional co-activators (p300/CBP) to HIF-1α (Figure 3) (Speer et al., 2013). Hydroxylation of HIF α subunit by PHDs is an evolutionarily conserved mechanism of sensing the oxygen concentration inside the cell. Thus, stabilization of HIF-1α by an exogenous technique may activate transcriptional and posttranscriptional responses that will help the cell to overcome hypoxic damage (Masoud and Li, 2015).

HIF-1α degradation can be restrained by inhibiting the activity of PHDs and FIH. PHDs and FIH belong to a class of enzymes known as 2-oxoglutarate dependent dioxygenases and are the most prominent known family of non-heme oxidizing enzymes (Schofield and Zhang, 1999). There are three PHD isoforms, PHD1, PHD2 and PHD3. Even though these isoforms have a similar C-terminal catalytic domain, N-terminal sequences differ significantly (Siddiq et al., 2009). mRNA expression analysis revealed that the expression patterns of PHD isoforms are tissue-specific and all the isoforms play a unique role in the regulation of HIF-1α and HIF-2α (Appelhoff et al., 2004). Both PHDs and FIH require oxygen, iron (Fe^2+^) and 2OG for the hydroxylation reaction (Singh et al., 2012).

Hypoxic microenvironments are important in the developing embryo, and they control cellular differentiation. These hypoxic microenvironments prompt the cells to activate HIFs which in turn regulate the development of the blood, vasculature, placenta, nervous system, and other organs (Simon and Keith, 2008). Thus, HIFs have a crucial role in embryonic development and survival. Validating the role of HIF during embryonic development, studies using HIF-1α deficient mouse embryos stopped developing by day 9 and died by day 10.5 due to extensive cell death and other abnormalities (Semenza, 2014).

## The Relevance of the HIF-1 Pathway in Ischemic Stroke

Cerebral stroke is a significant cause of morbidity and mortality worldwide (Kim and Johnston, 2013). More than 80% of strokes are ischemic while the remaining is hemorrhagic. Ischemic stroke occurs when a vessel supplying blood to the brain is obstructed due to a thrombus, embolus, or other blockage (Harten et al., 2010). Prevention of neuronal death and improving the recovery following ischemic injury are the central focuses of developing stroke therapeutics. Apart from tissue plasminogen activator (tPA), no drugs are able to confer neuroprotection following ischemic stroke in clinical studies (Shi, 2009).

A large number of preclinical studies that used PHD inhibitors were able to show that the activation of HIF-1 can be a potentially attractive way to achieve significant neuroprotection following ischemic injury (Karuppagounder and Ratan, 2012). Apart from stroke, PHD inhibition persistently extended neuroprotection in diverse neurological disease models (Speer et al., 2013). At the same time, a few studies observed adverse effects on brain cells upon treatment with PHD inhibitors mostly because of the activation of pro-death proteins like BNIP3 and NIX (Chen W. et al., 2009). As hypoxia induces HIF-1α expression, most hypoxia-mimetic agents up-regulate HIF-1α and orchestrate its downstream gene expression (Karuppagounder and Ratan, 2012). Neuroprotection *via* HIF-1α stabilization following ischemic stroke is still a controversial topic due to its link to a vast variety of genes that mediate both adaptive and pathological processes (Vangeison et al., 2008). Unlike PHD inhibitors, not many FIH inhibitors are discovered, and the possibility of attaining neuroprotection *via* FIH inhibition has yet to be fully explored.

The altered expression of HIF-1 and its downstream genes during hypoxia have been very well studied in both *in vitro* and *in vivo* models (Semenza, 2001). Several processes like angiogenesis and erythropoiesis are connected to HIF-1 stabilization. Apart from this, recent studies were able to deduce more novel phenomena and genes linked to the HIF-1 pathway (Valsecchi et al., 2011; Liu et al., 2012). In a transient global ischemia model, HIF-1 expression was enhanced as early as 1 h in the recovery phase, and this enhanced expression lasted for 7 days (Chavez and Lamanna, 2002). Besides, expression of HIF-1 regulated genes, EPO and GLUT1 were elevated during the reperfusion period. The above study also found that insulin-like growth factor-1 (IGF-1) was able to induce HIF-1 expression *in vitro* and *in vivo*.

HIF-2α has 48% structural similarity with HIF-1α, and it also activates HRE-dependent gene transcription (Hu et al., 2003). However, HIF-1α and HIF-2α have discrete transcriptional targets and perform non-redundant roles. Their expression patterns are tissue specific and the part played by these isoforms in tumor progression is contrasting (Ratcliffe, 2007). A study observed that HIF-1 mediated genes conferred brain hypoxia-induced tolerance rather than HIF-2 regulated genes (Bernaudin et al., 2002). They came to this conclusion as because HIF-2α levels did not increase significantly in neuronal cells following hypoxia. Further, this study showed that adrenomedullin, propyl 4-hydroxylase α, metallothionein 1 (MT-1), mitogen-activated protein kinase phosphatase 1(MKP-1), CUGBP Elav-Like Family Member (CELF), 12-lipoxygenase and carbonic anhydrase 1 (CAR-1) are regulated by hypoxia. HIF-1α was also found to bind to the caspase-3 gene promoter region specifically. Moreover, HIF-1α and procaspase-3 showed similar expression pattern following photothrombotic cerebral ischemia (Hoecke et al., 2007).

Hypoxia-inducible factor responsive genes perform a significant role in the ischemic preconditioning (IPC) induced neuroprotection (Jones and Bergeron, 2001; Valsecchi et al., 2011). Up-regulation of cytochrome P450 2C11 by HIF-1α has a role in the tolerance induced by hypoxic preconditioning (HPC) in astrocytes (Liu and Alkayed, 2005). 2-methoxyestradiol (2ME2) is a metabolite of estrogen and an inhibitor of HIF-1α (Zhou et al., 2008). A study by Zhou and co-workers showed that 2ME2 aggravated the CA1 hippocampal cell death after global ischemia in rats (Zhou et al., 2008). Glucose at a concentration of 25 mM up-regulated the expression of HIF-1α in rat primary cortical neurons exposed to hypoxia. Surprisingly, the study showed that along with hypoxia, a reducing environment is required for the stabilization of HIF-1α in neuronal cells (Guo et al., 2008). The above lab also found out that BCL2 interacting protein 3 (BNIP3) and BCL2 interacting protein 3 like (NIX/BNIP3L) play a role in the HIF-1α mediated neuroprotection during ischemia-reperfusion (Guo, 2017).

Sodium-calcium exchanger-1 (NCX1) helps in the maintenance of Na^+^ and Ca^2+^ homeostasis (Michel et al., 2014). In a recent study, it was demonstrated that NCX1 gene is controlled by HIF-1 and contributes to the HIF-1 mediated neuroprotection during brain preconditioning (Valsecchi et al., 2011). The mammalian target of rapamycin (mTOR) belongs to a lipid kinases family, and it is involved in cell growth, survival, and proliferation (Yoon, 2017). mTOR facilitated neuroprotection under ischemic conditions through the activation of HIF-1α, and by the regulation of VEGF expression and neuronal apoptosis in developing rat brain (Chen et al., 2012).

A few studies showed that deletion of PHD2 in neuronal cells has beneficial effects like improvement in histological and functional outcomes and neovascularisation through the promotion of HIF-VEGF axis (Li et al., 2016). Supporting this observation, knockdown of HIF-1α before oxidative stress aggravated the impairment of learning and memory in rat model (Yang et al., 2017).

The effect of HIF-1α stabilization is not much studied in glial cells as in neurons. In a study by Vangeison et al. (2008) explored the impact of knocking down HIF-1α specifically in astrocytes in neuron/astrocyte co-cultures exposed to hypoxia. Selective deletion of HIF-1α in astrocytes significantly protected neurons from hypoxia-induced neuronal death in co-cultures. Myeloid-specific knockout (KO) of HIF-1α resulted in faster behavioral recovery of mice subjected to MCAO (Bok et al., 2017). The HIF-1α KO mice had fewer infiltrating microglia and apoptotic neurons in the hippocampus following MCAO compared to its wild-type counterpart. The above group also observed that HIF-1α governs microglial phagocytosis and production of ROS and TNF-α under ischemic conditions. Sirtuin 1 (Sirt 1) has many vital roles in response to metabolic stress, especially cerebral ischemia (Koronowski and Perez-Pinzon, 2015). A study by Jablonska et al. (2016) observed that the presence of HIF-1α plays an essential role in hypoxia-induced Sirt1 expression in oligodendrocyte progenitor cell (OPC).

### The Pro and Anti-apoptotic Face of HIF-1α Stabilization in Ischemic Stroke

HIF-1 has both pro and anti-apoptotic features. It improved the expression of genes like EPO and VEGF that are linked to several pathways related to neuroprotection. However, it also participates in the pro-apoptotic process by stabilizing the tumor suppressor protein p53 during severe hypoxia (Fan et al., 2009). Further, it promoted cell necrosis in collaboration with calcium and calpain. It was found to increase blood-brain barrier (BBB) permeability and thus heighten brain oedema.

In agreement with the pro-apoptotic role of HIF-1, a few studies found that HIF-1deficiency can confer neuroprotection against acute hypoxia-induced cell death in mice (Helton et al., 2005). A similar trend was observed in a study by Chen C. et al. (2009) that used HIF-1α siRNA. The authors showed that HIF-1α siRNA treatment protected neurons from ischemic injury *in vivo* through the inhibition of VEGF and apoptosis-related proteins like p53 and Caspase-3 along with HIF-1α. The *c*-glycosylated flavone, vitexin (5, 7, 4-trihydroxyflavone-8-glucoside) is a HIF-1α inhibitor (Min et al., 2015). Inhibition of HIF-1α in the early stages of neonatal cerebral ischemia with vitexin conferred neuroprotection *in vivo*.

Further, Chen et al. (2008) observed that inhibition of HIF-1α by 2ME2 5min after the hypoxia-ischemia protected the neuronal cells in the neonatal rat. There was no significant reduction in infarct volume when the treatment with the 2ME2 was delayed for 3hrs. HIF-1 inhibition also protected BBB and reduced brain oedema. While, the treatment with HIF-1α stabilizer DMOG, increased BBB permeability and brain oedema. Additionally, Barteczek et al. (2016) showed that HIF-1 and HIF-2 are not needed for cell survival under hypoxic conditions. This study also showed that HIF-1/2 deficiency might protect neurons from early neuronal cell death and neurological impairment caused by ischemic stroke.

Additionally, Aminova et al. (2004) showed that the pro-death or anti-death role of HIF-1α in a cell depends on the type of death stimulus. HIF-1α overexpressing HT22 cells showed increased sensitivity to glutamate toxicity, but they were more resistant to cell death induced by camptothecin or tunicamycin or thapsigargin. When the level of p53 is low, hypoxia-induced HIF-1α triggers the transcriptional activation of adaptive genes. However, during severe and sustained hypoxia, p53 levels increase along with HIF-1α stabilization and these proteins together activate pathological genes such as BAX (Halterman et al., 1999).

Tricyclodecan-9-ylxanthogenate (D609) down-regulates HIF-1α similar to 2ME2. Treatment with D609 or 2ME2 reduced infarct volume and improved neuroscore in rats following MCAO (Chen et al., 2007). These HIF-1α inhibitors decreased the expression of VEGF, BNIP3 and cleaved caspase 3. According to a study carried out by Mayhan (1999), VEGF promoted the BBB permeability *via* a nitric oxide synthase/cGMP-dependent pathway. Since VEGF is directly controlled by HIF-1, the negative effect of HIF-1α in ischemic stroke may be linked to VEGF. Information from the above studies suggests that the positive or negative effects of HIF-1 depend on cell type and the severity of hypoxia. The nature of ischemic insult also plays a role in determining whether HIF-1α will advocate pro-death or pro-survival pathways (Shi, 2009).

### Biphasic Expression Pattern of HIF-1α Following Ischemic Injury

Biphasic expression pattern of HIF-1α was observed in some of the studies following *in vitro* and *in vivo* models of cerebral ischemia. A study by Liu et al. (2015) monitored the changes in the HIF-1α gene and protein expression at different time points in a middle cerebral artery occlusion (MCAO) model using an embolus (eMCAO). HIF-1α mRNA level gradually increased following ischemia and peaked at 6 h. After that, it slowly decreased and came to a low level at 24 h. A similar pattern of expression was observed from 24 to 168 h reaching the peak at 72 h. An exactly same trend was seen in the case of HIF-1α protein expression.

According to a study by Yeh et al. (2010), inhibition of HIF-1α in the early time point (0.5 h after ischemic stress) abated brain injury and oedema. While inhibiting HIF-1α at a later stage (8 h after ischemic injury) increased brain damage and decreased VEGF expression. Theoretically, HIF-1α induction at the early time points of ischemia activates apoptotic pathways, and at late time points, it promotes cell survival pathways. Comparable expression pattern of HIF-1α was observed in other study carried out by Baranova et al. (2007) in the MCAO model.

In neonatal rat brain, upon MCAO, HIF-1α protein expression was elevated immediately (0 h, without reperfusion) and peaked at 8 h, and then deteriorated at 24 h after reperfusion (Mu et al., 2003). VEGF protein expression was also similar to that of HIF-1α. This variation shows the biphasic nature of HIF-1 expression following cerebral ischemia in neonatal brain. When compared to the expression pattern in neuronal cells, similar alterations in expression of HIF-1α mRNA and protein were observed in microglia cells following exposure to hypoxic conditions (Wang et al., 2017). These studies have enhanced our understanding of the dual and contrasting roles of HIF-1α expression in neurons following ischemic stroke. To a certain extent, this biphasic expression pattern of HIF-1α will decide the time window of administration of drugs that act *via* HIF-1α stabilization for maximum protection.

## Hypoxia Mimetic Agents in Ischemic Stroke

The term “hypoxia mimetic agent” has been widely used to indicate biological or chemical molecules which are used to up-regulate HIF-1α expression. They do it either *via* PHDs inhibition or some other mechanisms. Here, in this review, HIF-1α stabilizers are broadly classified according to the involvement of PHDs in the stabilization process.

### HIF-1α Up-Regulation *via* Inhibition of PHD Pathway

Most of the HIF-1α up-regulators act *via* PHD inhibition. Currently, the following interventions are used stabilize HIF-1α and mimic hypoxic conditions *via* PHD inhibition: (a) reduce Fe^2+^ availability using iron chelators; (b) introduce metal ions that will compete with Fe^2+^; (c) use 2OG analogs. HIF-1α stabilizers that do not come under any of the above categories are discussed under a separate subheading.

#### Iron Chelators and Competitors

Iron chelators are small molecules that bind very tightly to iron and reduce the amount of free divalent iron available for PHD mediated HIF hydroxylation reaction (Peyssonnaux et al., 2008). Deferoxamine mesylate (DFO) is a widely used iron chelator that removes excess iron from the body (Fisher et al., 2013). Previously, it was thought that iron chelators prevent oxidative injury by obstructing hydroxyl radical formation but later studies revealed that iron chelators inhibit the action of PHDs and upregulate HIF-1 expression (Siddiq et al., 2005). Alternatively, iron competitors (mostly divalent metal ions) that compete with Fe^2+^ in the PHD mediated HIF-1 hydroxylation process can be used. Although there are several iron competitors like Ni^2+^ and Mn2^+^, Co^2+^ in the form of cobalt chloride (CoCl_2_) is the most widely used iron competitor for PHD inhibition (Fandrey et al., 2006). A recent study by Na et al. (2015) reported that Zn^2+^ could selectively inhibit PHD3 over PHD2.

A study by Zaman et al. (1999) was one of the first studies to explore HIF-1 mediated neuroprotection by iron chelators following hypoxic stress. In this study, iron chelators, DFO and mimosine (MIM) protected embryonic cortical neurons from glutathione depletion and oxidative stress-induced cell death at a concentration of 100 μM. Neuroprotective capabilities of these agents were also comparable to their ability to augment DNA binding of HIF-1. These compounds also up-regulated mRNA and protein expression of HIF-1 controlled genes. Likewise, cell death induced by depletion of glutathione was decreased significantly by treatment with CoCl_2_ at a concentration > 200 μM, a competitive inhibitor of iron. CoCl_2_ also elevated the HIF-1α protein expression.

Subsequently, several studies explored the ability of iron chelators for HIF-1 mediated neuroprotection against hypoxic injury. Siddiq et al. (2005) investigated the mechanism by which DFO offered protection in neuronal cells. In cortical neurons, HIF-1α was stabilized upon treatment with 100 μM DFO and protected cells from oxidative glutamate toxicity. Similarly, Prass et al. (2002) demonstrated the neuroprotective effect of DFO against OGD injury in purified cortical neurons. DFO at a concentration of 150 μM/L induced 47% reduction in cell death in primary cortical neurons subjected to OGD.

Compound A is a novel proprietary (Fibrogen Inc., United States) small molecule iron chelator. It stabilized HIF-1α in cortical neurons and increased the expression of HIF-1 targeted genes at a concentration of 40 μM (Siddiq et al., 2005). It also conferred protection against oxidative glutamate toxicity. In addition to its protective effect in *in vitro* studies, treatment with 100 mg/kg of compound A, stabilized HIF-1 in rat brain within 3h. It also reduced infarct volume by 67%, compared to control littermates. In hippocampal neurons from newborn mice, pretreatment with 10 mM/L DFO decreased OGD induced cell death by 45% compared to the control group. The protection conferred by DFO diminished upon cell’s transfection with anti-HIF-1α. This finding suggests that the protection by DFO is through HIF-1α induction (Hamrick et al., 2005).

Prolyl hydroxylases isoforms differ in their expression patterns, tissue distribution, subcellular localization, and their ability to hydroxylate HIF-1α (Siddiq et al., 2009). Based on these characteristics, Siddiq and co-workers hypothesized that the neuroprotection conferred by PHD inhibitors are PHD isoform-specific and independent of HIF-1. In this regard, they investigated the role of PHD and HIF isoforms in giving neuroprotection against normoxic oxidative death by the treatment of PHD inhibitors in primary rat neurons. When compared to a previous *in vitro* study carried out by Zaman et al. (1999), they observed something distinctive in this study. They noted that the prevention of normoxic oxidative neuronal death by DFO is through the inhibition of PHD1 and HIF-1α doesn’t have any role in this mechanism. They also noted that its HIF-2α, not HIF-1α controls the sensitivity to normoxic oxidative neuronal death.

In comparison to adult CNS, the neonatal brain is highly sensitive to the availability of essential substrates. Treatment with 60 mg/kg of CoCl_2_ and 200 mg/kg of DFO increased the level of HIF-1α protein in rat pups compared to the vehicle-injected controls (Bergeron et al., 2000). Compared with that in vehicle-injected controls, preconditioning with DFO and CoCl_2_ 24h before hypoxia-ischemia gave 56 and 75% protection, respectively.

Desferrioxamine promoted the binding of HIF-1 to DNA and transcription of EPO *in vivo*. There was a 20-fold increase in DNA binding of HIF-1 upon DFO application. The tolerance induced by DFO was time and dosage-dependent (Prass et al., 2002). Expression of GLUT1 protein was elevated in rat brain by the treatment of 300 mg/kg of DFO. Brain lesion area and whole brain cell loss reduced upon treatment with DFO. Notably, DFO treatment didn’t affect striatal lesion but provided a 20% reduction in cortical injury compared with the vehicle group. Likewise, there was a 62% decrease in thalamic shrinkage by the treatment of DFO in comparison with vehicle-treated animals. Neurological score and sensorimotor performances also improved upon treatment with DFO (Freret et al., 2006).

Deferasirox (DFR) is an orally administrated iron chelator and is in clinical use with high tolerance (Hatcher et al., 2009). As previously observed in the study by Siddiq et al. (2009); Zhao and Rempe (2011) also showed that neuroprotection conferred by DFO and DFR following MCAO does not require HIF-1α in mice. They also observed that both DFO and DFR do not induce expression of HIF-1 target genes.

2, 2′-dipyridyl (DP) is a liposoluble iron chelator, and it is used to regulate HIF-1α expression up. Experiments show that DP treatment reduced infarct volume and maximal infarct area and thus significantly decreased histological damage (Demougeot et al., 2004). There was a 42% reduction of infarct volume and a 25% decrease in the maximal infarct area upon treatment with DP. It protected both endothelial cells and neurons from ischemic injury. Besides, DP treatment in rats reduced ischemia-induced reactive oxygen species (ROS) production and suppressed the transformation of penumbra into infarct.

In chemical photothrombosis stroke models, DP treatment hindered the progression of infarct core and the surrounding regions (Hoecke et al., 2005). The density of apoptotic bodies reduced upon treatment with DP in both infarct core and surrounding pale region. Compared to the vehicle-treated animals, DP treatment decreased and limited the DNA fragmentation only to P1 region. Upon treatment with DP, there was a significant reduction in procaspase-9 cleavage in the P1 and P2 regions. Similarly, procaspase-3 cleavage was decreased in these regions. There was also a drop in cleaved caspase-3 in the entire infarct region upon treatment with DP *in vivo*.

Neuroprotection achieved by DP post-treatment was lower when compared with DP pretreatment (Baranova et al., 2007). Further, the protective effect produced through the administration of DP before or after MCAO was significantly attenuated but not completely abolished in neuron-specific HIF-1α-deficient mice. This shows the involvement of an alternative mechanism in giving neuroprotection upon DP treatment. Similarly, another study (Jones and Bergeron, 2001) observed that there was no change in expression of genes modulated by HIF-1 upon preconditioning with CoCl_2_ suggesting a surrogate process may be involved in the induction of tolerance by CoCl_2_ in the brain of newborn rats.

#### 2OG Analogs

Numerous hydroxybenzenes, hydroxybenzoic acids and analogous compounds have similar structural features as both 2-oxoglutarate and ascorbate. They can compete with these molecules and inhibit prolyl 4-hydroxylase enzymes (Majamaa et al., 1986). 10 μM dihydroxybenzoic acid (DHB) stabilized HIF-1α and increased the transcription of HIF-1 dependent genes. DHB conferred protection to cortical neurons exposed to oxidative glutamate toxicity (Siddiq et al., 2005).

*N*-oxalylglycine can inhibit most of the 2OG-dependent oxygenases and other enzymes (Al-Qahtani et al., 2015). Dimethyloxalylglycine (DMOG) is a readily cell permeable ester of *N*-oxalylglycine. DMOG has a similar structure to 2OG. It competes with PHD co-substrate, 2OG and prevents HIF-1α degradation (Siddiq et al., 2009; Nagel et al., 2011). Twenty four hour treatment with DMOG increased the protein expression of HIF-1α and mRNA levels of VEGF in cortical neurons growing under normoxic condition. Preconditioning with DMOG for 24 h before OGD significantly reduced OGD-induced cell death in cortical neuron cell. Similarly, post-treatment with DMOG also reduced cell death. This protection abrogated by the pretreatment of HIF-1α-shRNA showed the need of HIF-1α for achieving neuroprotection using PHD inhibitors (Ogle et al., 2012).

*In vivo* stabilization of HIF-1 was observed within 6 h of administration of DHB. There was a significant reduction of infarct volume upon DHB treatment compared to control (Siddiq et al., 2005). Studies in mice showed that intraperitoneal injection of DMOG increased the level of HIF-1α protein up to threefold in a time-dependent manner in mouse brain cortex. It also elevated the expression of HIF-1 controlled genes that regulate erythropoietin and pyruvate dehydrogenase kinase-1. Further, post-OGD treatment with DMOG reduced the infarct volume in an animal stroke model. In addition to this, it stimulated pro-apoptotic caspase-3 protein, curtailed behavioral deficits and decreased the loss of local blood flow in the middle cerebral artery territory. These attributes nullified upon inhibition of HIF-1α by Digoxin (Ogle et al., 2012).

In another study by Nagel et al. (2011), DMOG reduced ischemic injury and improved behavior and neuroscore after both permanent and transient MCAO in adult male Wistar rats. Regional cerebral blood flow also improved. There was also a reduction in the amount of BBB breakdown. A consistent increase in the expression of both mRNA and protein levels of VEGF and endothelial nitric oxide synthase observed after DMOG treatment. As seen in the case of DFO, a study by Siddiq and co-workers suggested that the neuroprotection exerted by DMOG and DHF does not require HIF-1α stabilization and instead inhibit the PHD1 isoform (Siddiq et al., 2005).

#### PHD Inhibitors With an Alternative Mechanism of Action

This section also includes HIF-1α stabilizers, but their exact mechanism of PHD inhibition is not either mentioned in the source paper or different from the above classification. These molecules may act by binding directly to the active site of the PHDs without mimicking 2OG or chelating the iron atom. A study (Reischl et al., 2014) focusing on reducing neuronal death following ischemic stroke *via* maintaining BBB integrity used a novel PHD inhibitor, FG-4497. HIF-1α stabilized upon treatment with FG-4497 in both primary murine astrocytes and the murine cerebrovascular endothelial cell line. FG-4497’s capability to inhibit HIF-1α proteolysis was higher than DMOG. It also up-regulated the transcription of HIF-1 targeted genes VEGF and EPO. In mouse hippocampal culture, FG-4497 significantly enhanced the survival of cells following OGD.

Intraperitoneal injection of FG-4497 elevated the amount of HIF-1α in cerebral tissue and significantly reduced infarct size compared to vehicle-treated mice (Reischl et al., 2014). FG-4497 pre-treatment reduces infarct volume in transient MCAO models. Post-treatment with FG-4497 also showed neuroprotection following permanent cerebral ischemia.

2-(1-chloro-4-hydroxyisoquinoline-3-carboxamido) acetic acid (IOX3) is a novel inhibitor of PHDs (Chen et al., 2014). It has a structure similar to FG2216, which promoted the expression of EPO *in vivo*. Administration of IOX3 24h before a MCAO improved neuroscores and reduced infarct volume in mice. It also elevated the level of HIF-1α protein and EPO expression. However, injection of IOX3 soon before the MCAO showed no significant neuroprotection. IOX3 also prevented BBB disruption when treated 24 h before MCAO.

GSK360A is an orally active PHD inhibitor that has reduced myocardial infarct size in the rat and murine heart (Ong et al., 2014). Treatment with GSK360A increased the plasma EPO and VEGF mRNA and protein levels (Zhou et al., 2017). When compared to the vehicle-treated group, pretreatment with GSK360A reduced post-stroke surgery neurological deficits, cognitive dysfunction and infarct volume 4 weeks after MCAO. Another novel PHD inhibitor, TM6008 decreased cell death and up-regulated HIF-1α levels *in vitro*. It also induced the protein expression of HIF-1 downstream genes, HO-1, EPO, and glucose transporter 3 (GLUT3) (Kontani et al., 2013).

Lately, molecular docking and simulation studies carried out in our lab found folic acid (FA) to be an ideal compound that binds to PHD2, FIH and pVHL alike at their HIF-1α binding region (Davis et al., 2018). *In vitro* experiments showed that post-ischemic treatment with FA up-regulated HIF-1 expression and its downstream genes. Further, FA conferred neuroprotection following OGD and promoted angiogenesis.

### HIF-1α Up-Regulation Without the Inhibition of PHDs

Non-PHD HIF-1α stabilizers relay on a mechanism other than the classical PHD-HIF pathway to up-regulates HIF-1α. Compared to the conventional PHD inhibitors, these molecules are newly discovered. HIF-1α stabilization through IPC is not discussed in this review as it is out of the scope of the title theme.

A study by Liu et al. (2015) showed that miR-335 is a direct regulator of HIF-1α, and has shown an inverse expression profile both *in vivo* and *in vitro* ischemic models. Treatment with miR-335 decreased infarct volume immediately after MCAO, and miR-335 inhibitor was found to be beneficial at 24 h after MCAO in a rat model. Thus, regulation of HIF-1α expression by miR-335 at various time points manipulated the genes required for adaption to hypoxia and ultimately resulted in the reduction of infarct volume.

Preconditioning with 1.5% isoflurane protected neurons from oxygen-glucose deprivation (OGD) injury by the induction of HIF-1α protein, and this activation is linked to the Erk1/2 pathway (Li et al., 2008). Further, a recent study by the above group showed the neuroprotection provided by isoflurane post-conditioning *via* HIF-1α stabilization (Li et al., 2012). Post-ischemic treatment with isoflurane reduced the infarct volume and improved neurological scores. Both post and pre-ischemic treatment with isoflurane treatment up-regulated iNOS mRNA level.

*N*-acetylcysteine (NAC) has various therapeutic properties, and it is an antioxidant (Dhouib et al., 2016). It is also protected the brain from ischemic injury in preclinical studies (Zhang Z. et al., 2014). A study by Zhang and colleagues observed that pretreatment with 150 mg/kg of NAC increased the protein levels of HIF-1α, EPO and GLUT3 in the ipsilateral hemispheres of rodents. The group also found out that HIF-1 is responsible for NAC mediated neuroprotection following MCAO. NAC treatment up-regulated heat shock protein 90 (Hsp90) expression and in turn interaction of Hsp90 to HIF-1.

A study by Badawi and Shi (2017) reported that 20S and 26S proteasomal pathways were involved in HIF-1α degradation in ischemic neurons. For the *in vitro* studies, MG-132, a proteasome inhibitor was used and it stabilized HIF-1α protein better than DMOG. A combination of MG-132 and DMOG had a better effect on HIF-1α stabilization than them individually. Further, they observed that treatment with epoxomicin, another proteasome inhibitor reduced infarct size and brain oedema following oxidative stress *in vivo* by the stabilization of HIF-1α. *In vivo* also a combination of epoxomicin and DMOG yielded better protection. The study also showed that proteasome inhibitors are more effective than PHD inhibitors in providing HIF-1α stabilization and neuroprotection.

Doeppner et al. (2012) tested a novel proteasome inhibitor BSc2118 for its neuroprotective activity following ischemic stroke. 12h pre-ischemic injection of BSc2118 conferred significant reduction of infarct volumes on day four. BSc2118 yielded long-term neuroprotection for as long as 3 months with the post-stroke treatment. Apart from reducing the infarct volume, BSc2118 was able to decrease brain oedema, and BBB break down. Further, it promoted both angiogenesis and neurogenesis. Using the knock out studies, they concluded that all these positive effects of BSc2118 were mediated by HIF-1α.

Tilorone is a low molecular weight antiviral agent (Ekins et al., 2017). In a study by Ratan et al. (2008) tilorone at a concentration of 100 mg/kg increased the stabilization of HIF-1α and elevated the expression of its downstream genes *in vitro*. The stabilization of HIF-1α by tilorone was independent of iron chelation and HIF-PHD inhibition *in vitro*. Tilorone also significantly reduced infarct volume *in vivo* following MCAO.

Huang-Lian-Jie-Du-Tang (HLJDT) is a traditional Chinese medicine with properties like heat-clearing and detoxification (Zhang Q. et al., 2014). In cerebral cortical neurons, pretreatment with HLJDT increased HIF-1α, EPO and VEGF levels and protected the cells against OGD. It decreased ischemia-induced apoptosis and promoted proliferation in neuronal cells. HLJDT also significantly reduced cerebral infarction; cerebral water content and improved neurological deficient score in MCAO rat models. All the hypoxia mimetic agents discussed in this review either act by inhibiting PHDs or other mechanisms and they ultimately regulate the expression of HIF-1. Tables 1, 2 gives a summary of the studies that used hypoxia-mimetic agents to obtain neuroprotection following ischemic injury.

**Table 1:** Details about the studies that used non-PHD HIF-1α stabilizers as hypoxia-mimetic agents.

Sl. No.	Study	Compound(s)	Type of compound	Time of administration	Stroke model(s)	Cell/animal type
1	Liu et al., 2015	miR-335	microRNA	Immediately or 24 h after MCAO	eMCAO	Male Wistar rats
2	Li et al., 2008	Isoflurane	Anesthetic	24 h before OGD	OGD	Hippocampal neurons
3	Li et al., 2012	Isoflurane		Not specified (post-OGD/MCAO)	OGD/MCAO	Male Sprague–Dawley rats; Primary cortical neurons
4	Zhang Z. et al., 2014	NAC	Antioxidant	30 min before MCAO	MCAO	Male Sprague–Dawley rats
5	Badawi and Shi, 2017	MG-132; Epoxomicin	Proteasome inhibitor	24 h before MCAO	OGD/MCAO	C57/BL/6 male mice; Primary cortical neurons
6	Doeppner et al., 2012	BSc2118	Proteasome inhibitor	12 h before MCAO	MCAO	Male C57BL/6N mice
7	Ratan et al., 2008	Tilorone	Antiviral agent	24 h before MCAO	MCAO	Male Sprague–Dawley rats

**Table 2:** Details about the studies that used PHD inhibitors as hypoxia-mimetic agents.

Sl. No.	Study	Compound(s)	Type of compound	Time of administration	Stroke model(s)	Cell/animal type
1	Zaman et al., 1999	DFO	Iron chelator	Immediately or up to 10 h after HCA^∗^ treatment	Glutathione depletion model	Rat primary neurons
		Mimosine	Iron chelator			
		CoCl_2_	Iron competitor			
2	Siddiq et al., 2005	DFO		6 h before MCAO	Glutamate-mediated excitotoxicity/MCAO	Rat primary neurons; Adult male Sprague–Dawley rats
		DHB	2OG analog			
		Compound A	Iron chelator			
3	Bergeron et al., 2000	DFO		24 h before ligation	Unilateral carotid artery ligation	Male and female Sprague–Dawley rats
		CoCl2				
4	Prass et al., 2002	DFO		48 or 72 h before OGD/MCAO	OGD/MCAO	Rat primary neurons; Male Wistar rats
5	Freret et al., 2006	DFO		Before and/or shortly after MCAO	MCAO	Sprague–Dawley rats
6	Baranova et al., 2007	DFO		6 h before or after MCAO	MCAO	Transgenic C57BL6/J male mice
		DHB				
		DP	Iron chelator			
7	Siddiq et al., 2009	DFO		Before or along with HCA	Glutathione depletion model	HT22 murine hippocampal cells
		DHB				
		DMOG	2OG analog			
8	Hamrick et al., 2005	DFO		1 h before and during OGD	OGD	Hippocampal cells
9	Mu et al., 2005	DFO		Immediately after MCAO	MCAO	Female Sprague–Dawley rats
10	Hanson et al., 2009	DFO		Multiple times before or after MCAO	MCAO	Male Sprague–Dawley rats
11	Zhao and Rempe, 2011	DFO		Every day up to 4 weeks before MCAO	MCAO	Mice
		DFR	Iron chelator			
12	Jones and Bergeron, 2001	CoCl_2_		24 h before MCAO	MCAO	Male and female Sprague–Dawley rats
13	Demougeot et al., 2004	DP	Iron chelator	15 min before and 1 h after occlusion	Cortical photothrombotic vascular occlusion	Adult male Wistar rats
14	Hoecke et al., 2005	DP		15 min before and 1 h after occlusion	Cortical photothrombotic vascular occlusion	Adult male Wistar rats
15	Ogle et al., 2012	DMOG		24 h before OGD and 2 h after initiation of OGD; 30 or 60 min after reperfusion	OGD/MCAO	Primary neurons; Adult male B6129PF2/J mice
16	Nagel et al., 2011	DMOG		Multiple times before and one time after MCAO	Permanent or transient MCAO	Adult male Wistar rats
17	Reischl et al., 2014	FG-4497	Not specified	6, 24, and 48 h during OGD; 6 h before or soon after MCAO	MCAO	Primary murine astrocytes and HT-22 hippocampal neuronal cells; Male C57BL/6 mice
18	Chen et al., 2014	IOX3	Not specified	One day or immediately before MCAO	MCAO	Male C57/B6 mice
19	Zhou et al., 2017	GSK360A	Not specified	18 and 5 h before MCAO	MCAO	Male Sprague–Dawley rats
20	Kontani et al., 2013	TM6008	Not specified	Immediately after hypoxia	Anaerobic chamber	SHSY-5Y cells
21	Zhang Q. et al., 2014	HLJDT	Unknown	24 h before OGD or MCAO	OGD; MCAO	Rat primary neurons; Male Sprague–Dawley rats

*^∗^HCA, homocysteic acid.*

## Conclusion

Hypoxia causes the cell to accumulate HIF-1 in the nucleus and up-regulate specific HIF-1 targeted genes so that cells can overcome the adverse condition. All the hypoxia mimetic agents are designed to act in such a way to boost this endogenous mechanism. One way to do this is through the inhibition of PHDs. These PHD inhibitors come under either the iron chelator and competitive inhibitor category or the 2OG analog category. Other molecules rely on mechanisms other than PHD inhibition to stabilize HIF-1α. Apart from a few newly discovered molecules, widely used hypoxia-mimetic agents are DFO, DHB, CoCl_2_, DP, and DMOG. These agents up-regulated HIF-1α levels and regulated its downstream genes.

Further, these agents conferred significant neuroprotection both *in vitro* and *in vivo* experiments. As the progression of ischemic brain injury is multifactorial, each of the HIF-1 regulated genes influences various cellular processes of infarct advancement at different time points. In most of the cases, treatment with hypoxia-mimetic agents several hours before or after the induction of hypoxia bestowed greater protection compared to treatment with these agents soon before or after oxidative stress. The neuroprotection conferred by these agents also depends on the cell type and magnitude of the ischemic injury.

However, there are studies which did not show neuroprotection *via* HIF-1 up-regulation and even demonstrated its pro-death features. Therefore, the extent of neuroprotection provided by HIF-1 in the case of ischemic injury is still a debatable topic. Nevertheless, in light of studies discussed in this review (Tables 1, 2); we can conclude that HIF-1’s beneficial characteristics appear to outweigh its adverse effects. Many of the harmful effects of HIF-1α can be eliminated by carefully choosing the time of administration of HIF-1α stabilizers. Type of hypoxia mimetic agent used, nature of ischemic injury and kind of cell targeted by the treatment are the other factors which affect the neuroprotection bestowed by HIF-1α stabilizers.

One drawback of using the conventional mimetic agent is that several other enzymes use Fe^2+^ and 2OG as co-factor or co-substrate. Therefore the use of iron chelators, competitive iron inhibitors and 2OG analogs can create undesired interference of different pathways. In this regard, the introductions of novel drugs that can specifically inhibit PHDs or stabilize HIF-1α through other mechanisms need more consideration. Specificity of PHD inhibitors can be achieved by the development of molecules that can bind to the active sites in PHDs. These agents could be analogous to the parts of HIF-1α that interact with PHDs. The possibility of stabilizing HIF-1α *via* FIH inhibition has not received much attention and warrants further exploration.

## Author Contributions

GR and AM conceived the idea. CD wrote the manuscript. SJ, GR, AM, and O-NB helped to draft the manuscript.

## Conflict of Interest Statement

The authors declare that the research was conducted in the absence of any commercial or financial relationships that could be construed as a potential conflict of interest.

## References

[B1] Al-Qahtani K. Jabeen B. Sekirnik R. Riaz N. Claridge T. D. Schofield C. J. (2015). The broad spectrum 2-oxoglutarate oxygenase inhibitor N-oxalylglycine is present in rhubarb and spinach leaves. *Phytochemistry* 117 456–461. 10.1016/j.phytochem.2015.06.028 26196940

[B2] Aminova L. R. Chavez J. C. Lee J. Ryu H. Kung A. Lamanna J. C. (2004). Prosurvival and prodeath effects of hypoxia-inducible factor-1α stabilization in a murine hippocampal cell line. *J. Biol. Chem.* 280 3996–4003. 10.1074/jbc.m409223200 15557337

[B3] Appelhoff R. J. Tian Y.-M. Raval R. R. Turley H. Harris A. L. Pugh C. W. (2004). Differential function of the Prolyl Hydroxylases PHD1, PHD2, and PHD3 in the regulation of hypoxia-inducible factor. *J. Biol. Chem.* 279 38458–38465. 10.1074/jbc.m406026200 15247232

[B4] Badawi Y. Shi H. (2017). Relative contribution of rolyl Hydroxylase-dependent and -independent degradation of HIF-1alpha by proteasomal pathways in cerebral ischemia. *Front. Neurosci.* 11:239. 10.3389/fnins.2017.00239 28566998 PMC5434458

[B5] Baranova O. Miranda L. F. Pichiule P. Dragatsis I. Johnson R. S. Chavez J. C. (2007). Neuron-specific inactivation of the hypoxia inducible factor 1 increases brain injury in a mouse model of transient focal cerebral ischemia. *J. Neurosci.* 27 6320–6332. 10.1523/jneurosci.0449-07.2007 17554006 PMC6672155

[B6] Barteczek P. Li L. Ernst A.-S. Böhler L.-I. Marti H. H. Kunze R. (2016). Neuronal HIF-1α and HIF-2α deficiency improves neuronal survival and sensorimotor function in the early acute phase after ischemic stroke. *J. Cereb. Blood Flow Metab.* 37 291–306. 10.1177/0271678x15624933 26746864 PMC5363746

[B7] Beaudry M. Hidalgo M. Launay T. Bello V. Darribère T. (2016). Regulation of myogenesis by environmental hypoxia. *J. Cell Sci.* 129 2887–2896. 10.1242/jcs.188904 27505427

[B8] Bergeron M. Gidday J. M. Yu A. Y. Semenza G. L. Ferriero D. M. Sharp F. R. (2000). Role of hypoxia-inducible factor-1 in hypoxia-induced ischemic tolerance in neonatal rat brain. *Ann. Neurol.* 48 285–296. 10.1002/1531-8249(200009)48:3<285::AID-ANA2>3.0.CO;2-8 10976634

[B9] Bernaudin M. Tang Y. Reilly M. Petit E. Sharp F. R. (2002). Brain genomic response following hypoxia and re-oxygenation in the neonatal rat. *J. Biol. Chem.* 277 39728–39738. 10.1074/jbc.m204619200 12145288

[B10] Bok S. Kim Y.-E. Woo Y. Kim S. Kang S.-J. Lee Y. (2017). Hypoxia-inducible factor-1α regulates microglial functions affecting neuronal survival in the acute phase of ischemic stroke in mice. *Oncotarget* 8 111508–111521. 10.18632/oncotarget.22851 29340071 PMC5762339

[B11] Chavez J. C. Lamanna J. C. (2002). Activation of hypoxia-inducible factor-1 in the rat cerebral cortex after transient global ischemia: potential role of insulin-like growth factor-1. *J. Neurosci.* 22 8922–8931. 10.1523/jneurosci.22-20-08922.2002 12388599 PMC6757701

[B12] Chen C. Hu Q. Yan J. Lei J. Qin L. Shi X. (2007). Multiple effects of 2ME2 and D609 on the cortical expression of HIF-1α and apoptotic genes in a middle cerebral artery occlusion induced focal ischemia rat model. *J. Neurochem.* 102 1831–1841. 10.1111/j.1471-4159.2007.004652.x 17532791

[B13] Chen C. Hu Q. Yan J. Yang X. Shi X. Lei J. (2009). Early inhibition of HIF-1α with small interfering RNA reduces ischemic–reperfused brain injury in rats. *Neurobiol. Dis.* 33 509–517. 10.1016/j.nbd.2008.12.010 19166937

[B14] Chen H. Xiong T. Qu Y. Zhao F. Ferriero D. Mu D. (2012). mTOR activates hypoxia-inducible factor-1α and inhibits neuronal apoptosis in the developing rat brain during the early phase after hypoxia–ischemia. *Neurosci. Lett.* 507 118–123. 10.1016/j.neulet.2011.11.058 22178140 PMC3525671

[B15] Chen R. L. Ogunshola O. O. Yeoh K. K. Jani A. Papadakis M. Nagel S. (2014). HIF prolyl hydroxylase inhibition prior to transient focal cerebral ischaemia is neuroprotective in mice. *J. Neurochem.* 131 177–189. 10.1111/jnc.12804 24974727

[B16] Chen W. Jadhav V. Tang J. Zhang J. H. (2008). HIF-1 alpha inhibition ameliorates neonatal brain damage after hypoxic-ischemic injury. *Acta Neurochir. Suppl.* 102 395–399. 10.1007/978-3-211-85578-2_77 19388354

[B17] Chen W. Ostrowski R. P. Obenaus A. Zhang J. H. (2009). Prodeath or prosurvival: two facets of hypoxia inducible factor-1 in perinatal brain injury. *Exp. Neurol.* 216 7–15. 10.1016/j.expneurol.2008.10.016 19041643 PMC2672430

[B18] Davis C. K. Nampoothiri S. S. Rajanikant G. K. (2018). Folic acid exerts post-ischemic neuroprotection *in vitro* through HIF-1α stabilization. *Mol. Neurobiol.* 55 8328–8345. 10.1007/s12035-018-0982-3 29542054

[B19] Demougeot C. Van Hoecke M. Bertrand N. Prigent-Tessier A. Mossiat C. Beley A. (2004). Cytoprotective efficacy and mechanisms of the liposoluble iron chelator 2, 2’-dipyridyl in the rat photothrombotic ischemic stroke model. *J. Pharmacol. Exp. Ther.* 311 1080–1087. 10.1124/jpet.104.072744 15280435

[B20] Dhouib I. E. Jallouli M. Annabi A. Gharbi N. Elfazaa S. Lasram M. M. (2016). A minireview on N -acetylcysteine: an old drug with new approaches. *Life Sci.* 151 359–363. 10.1016/j.lfs.2016.03.003 26946308

[B21] Doeppner T. R. Mlynarczuk-Bialy I. Kuckelkorn U. Kaltwasser B. Herz J. Hasan M. R. (2012). The novel proteasome inhibitor BSc2118 protects against cerebral ischaemia through HIF1A accumulation and enhanced angioneurogenesis. *Brain* 135 3282–3297. 10.1093/brain/aws269 23169919

[B22] Ekins S. Lingerfelt M. A. Comer J. E. Freiberg A. N. Mirsalis J. C. Oloughlin K. (2017). Efficacy of tilorone dihydrochloride against ebola virus infection. *Antimicrob. Agents Chemother.* 62 e1711–e1717. 10.1128/aac.01711-17 29133569 PMC5786809

[B23] Fan X. Heijnen C. J. Kooij M. A. V. D. Groenendaal F. Bel F. V. (2009). The role and regulation of hypoxia-inducible factor-1α expression in brain development and neonatal hypoxic–ischemic brain injury. *Brain Res. Rev.* 62 99–108. 10.1016/j.brainresrev.2009.09.006 19786048

[B24] Fandrey J. Gorr T. Gassmann M. (2006). Regulating cellular oxygen sensing by hydroxylation. *Cardiovasc. Res.* 71 642–651. 10.1016/j.cardiores.2006.05.005 16780822

[B25] Fisher S. A. Brunskill S. J. Doree C. Gooding S. Chowdhury O. Roberts D. J. (2013). Desferrioxamine mesylate for managing transfusional iron overload in people with transfusion-dependent thalassaemia. *Cochrane Database Syst. Rev.* 21:CD004450. 10.1002/14651858.cd004450.pub3 23963793 PMC11491190

[B26] Freret T. Valable S. Chazalviel L. Saulnier R. Mackenzie E. T. Petit E. (2006). Delayed administration of deferoxamine reduces brain damage and promotes functional recovery after transient focal cerebral ischemia in the rat. *Eur. J. Neurosci.* 23 1757–1765. 10.1111/j.1460-9568.2006.04699.x 16623832

[B27] Guo S. Bragina O. Xu Y. Cao Z. Chen H. Zhou B. (2008). Glucose up-regulates HIF-1α expression in primary cortical neurons in response to hypoxia through maintaining cellular redox status. *J. Neurochem.* 105 1849–1860. 10.1111/j.1471-4159.2008.05287.x 18266932 PMC12494482

[B28] Guo Y. (2017). Role of HIF-1a in regulating autophagic cell survival during cerebral ischemia reperfusion in rats. *Oncotarget* 8 98482–98494. 10.18632/oncotarget.21445 29228704 PMC5716744

[B29] Halterman M. W. Miller C. C. Federoff H. J. (1999). Hypoxia-inducible factor-1α mediates hypoxia-induced delayed neuronal death that involves p53. *J. Neurosci.* 19 6818–6824. 10.1523/jneurosci.19-16-06818.1999 10436039 PMC6782875

[B30] Hamrick S. E. G. Mcquillen P. S. Jiang X. Mu D. Madan A. Ferriero D. M. (2005). A role for hypoxia-inducible factor-1α in desferoxamine neuroprotection. *Neurosci. Lett.* 379 96–100. 10.1016/j.neulet.2004.12.080 15823423

[B31] Hanson L. R. Roeytenberg A. Martinez P. M. Coppes V. G. Sweet D. C. Rao R. J. (2009). Intranasal deferoxamine provides increased brain exposure and significant protection in rat ischemic stroke. *J. Pharmacol. Exp. Ther.* 330 679–686. 10.1124/jpet.108.149807 19509317 PMC2729791

[B32] Harten S. K. Ashcroft M. Maxwell P. H. (2010). Prolyl hydroxylase domain inhibitors: a route to HIF activation and neuroprotection. *Antioxid. Redox Signal.* 12 459–480. 10.1089/ars.2009.2870 19737089

[B33] Hatcher H. C. Singh R. N. Torti F. M. Torti S. V. (2009). Synthetic and natural iron chelators: therapeutic potential and clinical use. *Future Med. Chem.* 1 1643–1670. 10.4155/fmc.09.121 21425984 PMC3821171

[B34] Helton R. Cui J. Scheel J. R. Ellison J. A. Ames C. Gibson C. (2005). Brain-specific knock-out of hypoxia-inducible factor-1 reduces rather than increases hypoxic-ischemic damage. *J. Neurosci.* 25 4099–4107. 10.1523/jneurosci.4555-04.2005 15843612 PMC6724950

[B35] Hoecke M. V. Prigent-Tessier A. Bertrand N. Prevotat L. Marie C. Beley A. (2005). Apoptotic cell death progression after photothrombotic focal cerebral ischaemia: effects of the lipophilic iron chelator 2,2′-dipyridyl. *Eur. J. Neurosci.* 22 1045–1056. 10.1111/j.1460-9568.2005.04297.x 16176346

[B36] Hoecke M. V. Prigent-Tessier A. S. Garnier P. E. Bertrand N. M. Filomenko R. Bettaieb A. (2007). Evidence of HIF-1 functional binding activity to caspase-3 promoter after photothrombotic cerebral ischemia. *Mol. Cell. Neurosci.* 34 40–47. 10.1016/j.mcn.2006.09.009 17101276

[B37] Hu C.-J. Wang L.-Y. Chodosh L. A. Keith B. Simon M. C. (2003). Differential roles of Hypoxia-Inducible Factor 1 (HIF-1) and HIF-2 in hypoxic gene regulation. *Mol. Cell. Biol.* 23 9361–9374. 10.1128/mcb.23.24.9361-9374.2003 14645546 PMC309606

[B38] Jablonska B. Gierdalski M. Chew L.-J. Hawley T. Catron M. Lichauco A. (2016). Sirt1 regulates glial progenitor proliferation and regeneration in white matter after neonatal brain injury. *Nat. Commun.* 7:13866. 10.1038/ncomms13866 27991597 PMC5187440

[B39] Jones N. M. Bergeron M. (2001). Hypoxic preconditioning induces changes in HIF-1 target genes in neonatal rat brain. *J. Cereb. Blood Flow Metab.* 21 1105–1114. 10.1097/00004647-200109000-00008 11524615

[B40] Karuppagounder S. S. Ratan R. R. (2012). Hypoxia-inducible factor prolyl hydroxylase inhibition: robust new target or another big bust for stroke therapeutics? *J. Cereb. Blood Flow Metab.* 32 1347–1361. 10.1038/jcbfm.2012.28 22415525 PMC3390817

[B41] Kim A. S. Johnston S. C. (2013). Temporal and geographic trends in the global stroke epidemic. *Stroke* 44 S123–S125. 10.1161/strokeaha.111.000067 23709707

[B42] Kontani S. Nagata E. Uesugi T. Moriya Y. Fujii N. Miyata T. (2013). A novel prolyl hydroxylase inhibitor protects against cell death after hypoxia. *Neurochem. Res.* 38 2588–2594. 10.1007/s11064-013-1175-0 24132642 PMC3898357

[B43] Koronowski K. Perez-Pinzon M. (2015). Sirt1 in cerebral ischemia. *Brain Circ.* 1 69–78. 10.4103/2394-8108.162532 26819971 PMC4725589

[B44] Li L. Saliba P. Reischl S. Marti H. H. Kunze R. (2016). Neuronal deficiency of HIF prolyl 4-hydroxylase 2 in mice improves ischemic stroke recovery in an HIF dependent manner. *Neurobiol. Dis.* 91 221–235. 10.1016/j.nbd.2016.03.018 27001147

[B45] Li Q. F. Xu H. Sun Y. Hu R. Jiang H. (2012). Induction of inducible nitric oxide synthase by isoflurane post-conditioning via hypoxia inducible factor-1α during tolerance against ischemic neuronal injury. *Brain Res.* 1451 1–9. 10.1016/j.brainres.2012.02.055 22445062

[B46] Li Q.-F. Zhu Y.-S. Jiang H. (2008). Isoflurane preconditioning activates HIF-1α, iNOS and Erk1/2 and protects against oxygen–glucose deprivation neuronal injury. *Brain Res.* 1245 26–35. 10.1016/j.brainres.2008.09.069 18930717

[B47] Liu F. J. Kaur P. Karolina D. S. Sepramaniam S. Armugam A. Wong P. T. H. (2015). MiR-335 regulates Hif-1α to reduce cell death in both mouse cell line and rat ischemic models. *PLoS One* 10:e0128432. 10.1371/journal.pone.0128432 26030758 PMC4452242

[B48] Liu M. Alkayed N. J. (2005). Hypoxic preconditioning and tolerance via Hypoxia Inducible Factor (HIF) 1α-linked induction of P450 2C11 epoxygenase in astrocytes. *J. Cereb. Blood Flow Metab.* 25 939–948. 10.1038/sj.jcbfm.9600085.a 15729289

[B49] Liu W. Shen S.-M. Zhao X.-Y. Chen G.-Q. (2012). Targeted genes and interacting proteins of hypoxia inducible factor-1. *Int. J. Biochem. Mol. Biol.* 3 165–178. 22773957 PMC3388736

[B50] Majamaa K. Günzler V. Hanauske-Abel H. M. Myllylä R. Kivirikko K. I. (1986). Partial identity of the 2-oxoglutarate and ascorbate binding sites of prolyl 4-hydroxylase. *J. Biol. Chem.* 261 7819–7823. 3011801

[B51] Masoud G. N. Li W. (2015). HIF-1α pathway: role, regulation and intervention for cancer therapy. *Acta Pharm. Sin. B* 5 378–389. 10.1016/j.apsb.2015.05.007 26579469 PMC4629436

[B52] Mayhan W. G. (1999). VEGF increases permeability of the blood-brain barrier via a nitric oxide synthase/cGMP-dependent pathway. *Am. J. Physiol Cell Physiol.* 276 C1148–C1153. 10.1152/ajpcell.1999.276.5.c1148 10329964

[B53] Michel L. Y. M. Verkaart S. Koopman W. J. H. Willems P. H. G. M. Hoenderop J. G. J. Bindels R. J. M. (2014). Function and regulation of the Na -Ca2 exchanger NCX3 splice variants in brain and skeletal muscle. *J. Biol. Chem.* 289 11293–11303. 10.1074/jbc.m113.529388 24616101 PMC4036267

[B54] Min J.-W. Hu J.-J. He M. Sanchez R. M. Huang W.-X. Liu Y.-Q. (2015). Vitexin reduces hypoxia–ischemia neonatal brain injury by the inhibition of HIF-1alpha in a rat pup model. *Neuropharmacology* 99 38–50. 10.1016/j.neuropharm.2015.07.007 26187393

[B55] Mu D. Jiang X. Sheldon R. Fox C. K. Hamrick S. E. Vexler Z. S. (2003). Regulation of hypoxia-inducible factor 1α and induction of vascular endothelial growth factor in a rat neonatal stroke model. *Neurobiol. Dis.* 14 524–534. 10.1016/j.nbd.2003.08.020 14678768

[B56] Mu D. Chang Y. S. Vexler Z. S. Ferriero D. M. (2005). Hypoxia-inducible factor 1α and erythropoietin upregulation with deferoxamine salvage after neonatal stroke. *Exp. Neurol.* 195 407–415. 10.1016/j.expneurol.2005.06.001 16023639

[B57] Na Y.-R. Woo D. J. Choo H. Chung H. S. Yang E. G. (2015). Selective inhibition of the hypoxia-inducible factor prolyl hydroxylase PHD3 by Zn(ii). *Chem. Commun.* 51 10730–10733. 10.1039/c5cc02143j 26051901

[B58] Nagel S. Papadakis M. Chen R. Hoyte L. C. Brooks K. J. Gallichan D. (2011). Neuroprotection by dimethyloxalylglycine following permanent and transient focal cerebral ischemia in rats. *J. Cereb. Blood Flow Metab.* 31 132–143. 10.1038/jcbfm.2010.60 20407463 PMC3049478

[B59] Ogle M. E. Gu X. Espinera A. R. Wei L. (2012). Inhibition of prolyl hydroxylases by dimethyloxaloylglycine after stroke reduces ischemic brain injury and requires hypoxia inducible factor-1α. *Neurobiol. Dis.* 45 733–742. 10.1016/j.nbd.2011.10.020 22061780 PMC3286647

[B60] Ong S.-G. Lee W. H. Theodorou L. Kodo K. Lim S. Y. Shukla D. H. (2014). HIF-1 reduces ischaemia–reperfusion injury in the heart by targeting the mitochondrial permeability transition pore. *Cardiovasc. Res.* 104 24–36. 10.1093/cvr/cvu172 25063991

[B61] Peyssonnaux C. Nizet V. Johnson R. S. (2008). Role of the hypoxia inducible factors HIF in iron metabolism. *Cell Cycle* 7 28–32. 10.4161/cc.7.1.5145 18212530

[B62] Prass K. Ruscher K. Karsch M. Isaev N. Megow D. Priller J. (2002). Desferrioxamine induces delayed tolerance against cerebral ischemia *in vivo* and *in vitro*. *J. Cereb. Blood Flow Metab.* 22 520–525. 10.1097/00004647-200205000-00003 11973424

[B63] Ratan R. R. Siddiq A. Aminova L. Langley B. Mcconoughey S. Karpisheva K. (2008). Small molecule activation of adaptive gene expression. *Ann. N. Y. Acad. Sci.* 1147 383–394. 10.1196/annals.1427.033 19076458 PMC2921907

[B64] Ratcliffe P. J. (2007). HIF-1 and HIF-2: working alone or together in hypoxia? *J. Clin. Investig.* 117 862–865. 10.1172/jci31750 17404612 PMC1838952

[B65] Reischl S. Li L. Walkinshaw G. Flippin L. A. Marti H. H. Kunze R. (2014). Inhibition of HIF prolyl-4-hydroxylases by FG-4497 reduces brain tissue injury and edema formation during ischemic stroke. *PLoS One* 9:e84767. 10.1371/journal.pone.0084767 24409307 PMC3883663

[B66] Schofield C. J. Ratcliffe P. J. (2004). Oxygen sensing by HIF hydroxylases. *Nat. Rev. Mol. Cell Biol.* 5 343–354. 10.1038/nrm1366 15122348

[B67] Schofield C. J. Zhang Z. (1999). Structural and mechanistic studies on 2-oxoglutarate-dependent oxygenases and related enzymes. *Curr. Opin. Struct. Biol.* 9 722–731. 10.1016/s0959-440x(99)00036-6 10607676

[B68] Semenza G. L. (1998). Hypoxia-inducible factor 1: master regulator of O2 homeostasis. *Curr. Opin. Genet. Dev.* 8 588–594. 10.1016/s0959-437x(98)80016-6 9794818

[B69] Semenza G. L. (2000). HIF-1: mediator of physiological and pathophysiological responses to hypoxia. *J. Appl. Physiol.* 88 1474–1480. 10.1152/jappl.2000.88.4.1474 10749844

[B70] Semenza G. L. (2001). Hypoxia-inducible factor 1: oxygen homeostasis and disease pathophysiology. *Trends Mol. Med.* 7 345–350. 10.1016/s1471-4914(01)02090-1 11516994

[B71] Semenza G. L. (2014). Hypoxia-inducible factor 1 and cardiovascular disease. *Annu. Rev. Physiol.* 76 39–56. 10.1146/annurev-physiol-021113-170322 23988176 PMC4696033

[B72] Shi H. (2009). Hypoxia inducible factor 1 as a therapeutic target in ischemic stroke. *Curr. Med. Chem.* 16 4593–4600. 10.2174/092986709789760779 19903149 PMC2819104

[B73] Siddiq A. Aminova L. R. Troy C. M. Suh K. Messer Z. Semenza G. L. (2009). Selective inhibition of Hypoxia-Inducible Factor (HIF) prolyl-hydroxylase 1 mediates neuroprotection against normoxic oxidative death via HIF- and CREB-independent pathways. *J. Neurosci.* 29 8828–8838. 10.1523/jneurosci.1779-09.2009 19587290 PMC3290095

[B74] Siddiq A. Ayoub I. A. Chavez J. C. Aminova L. Shah S. Lamanna J. C. (2005). Hypoxia-inducible factor prolyl 4-hydroxylase inhibition. *J. Biol. Chem.* 280 41732–41743. 10.1074/jbc.m504963200 16227210 PMC2586128

[B75] Simon M. C. Keith B. (2008). The role of oxygen availability in embryonic development and stem cell function. *Nat. Rev. Mol. Cell Biol.* 9 285–296. 10.1038/nrm2354 18285802 PMC2876333

[B76] Singh N. Sharma G. Mishra V. Raghubir R. (2012). Hypoxia inducible factor-1: its potential role in cerebral ischemia. *Cell. Mol. Neurobiol.* 32 491–507. 10.1007/s10571-012-9803-9 22297543 PMC11498632

[B77] Speer R. E. Karuppagounder S. S. Basso M. Sleiman S. F. Kumar A. Brand D. (2013). Hypoxia-inducible factor prolyl hydroxylases as targets for neuroprotection by “antioxidant” metal chelators: from ferroptosis to stroke. *Free Radic. Biol. Med.* 62 26–36. 10.1016/j.freeradbiomed.2013.01.026 23376032 PMC4327984

[B78] Vadlapatla R. Vadlapudi A. Mitra A. (2013). Hypoxia-Inducible Factor-1 (HIF-1): a potential target for intervention in ocular neovascular diseases. *Curr. Drug Targets* 14 919–935. 10.2174/13894501113149990015 23701276 PMC4407697

[B79] Valsecchi V. Pignataro G. Prete A. D. Sirabella R. Matrone C. Boscia F. (2011). NCX1 Is a novel target gene for hypoxia-inducible factor-1 in ischemic brain preconditioning. *Stroke* 42 754–763. 10.1161/strokeaha.110.597583 21293012

[B80] Vangeison G. Carr D. Federoff H. J. Rempe D. A. (2008). The good, the bad, and the cell type-specific roles of hypoxia inducible factor-1 in neurons and astrocytes. *J. Neurosci.* 28 1988–1993. 10.1523/jneurosci.5323-07.2008 18287515 PMC6671445

[B81] Wang X. Ma J. Fu Q. Zhu L. Zhang Z. Zhang F. (2017). Role of hypoxia-inducible factor-1α in autophagic cell death in microglial cells induced by hypoxia. *Mol. Med. Rep.* 15 2097–2105. 10.3892/mmr.2017.6277 28259912 PMC5365019

[B82] Yang Y. Ju J. Deng M. Wang J. Liu H. Xiong L. (2017). Hypoxia inducible factor 1α promotes endogenous adaptive response in rat model of chronic cerebral hypoperfusion. *Int. J. Mol. Sci.* 18:3. 10.3390/ijms18010003 28106731 PMC5297638

[B83] Yeh S.-H. Ou L.-C. Gean P.-W. Hung J.-J. Chang W.-C. (2010). Selective inhibition of early-but not late-expressed HIF-1α is neuroprotective in rats after focal ischemic brain damage. *Brain Pathol.* 21 249–262. 10.1111/j.1750-3639.2010.00443.x 21029239 PMC8094320

[B84] Yoon M.-S. (2017). The role of mammalian target of rapamycin (mTOR) in insulin signaling. *Nutrients* 9:1176. 10.3390/nu9111176 29077002 PMC5707648

[B85] Zaman K. Ryu H. Hall D. Odonovan K. Lin K.-I. Miller M. P. (1999). Protection from oxidative stress–induced apoptosis in cortical neuronal cultures by iron chelators is associated with enhanced DNA binding of hypoxia-inducible factor-1 and ATF-1/CREB and increased expression of glycolytic enzymes, p21waf1/cip1, and erythropoietin. *J. Neurosci.* 19 9821–9830. 10.1523/jneurosci.19-22-09821.1999 10559391 PMC6782985

[B86] Zeng H.-L. Zhong Q. Qin Y.-L. Bu Q.-Q. Han X.-A. Jia H.-T. (2011). Hypoxia-mimetic agents inhibit proliferation and alter the morphology of human umbilical cord-derived mesenchymal stem cells. *BMC Cell Biol.* 12:32. 10.1186/1471-2121-12-32 21827650 PMC3166919

[B87] Zhang Q. Bian H. Li Y. Guo L. Tang Y. Zhu H. (2014). Preconditioning with the traditional Chinese medicine Huang-Lian-Jie-Du-Tang initiates HIF-1α-dependent neuroprotection against cerebral ischemia in rats. *J. Ethnopharmacol.* 154 443–452. 10.1016/j.jep.2014.04.022 24751364

[B88] Zhang Z. Yan J. Taheri S. Liu K. J. Shi H. (2014). Hypoxia-inducible factor 1 contributes to N-acetylcysteine’s protection in stroke. *Free Radic. Biol. Med.* 68 8–21. 10.1016/j.freeradbiomed.2013.11.007 24296245 PMC3943875

[B89] Zhao Y. Rempe D. A. (2011). Prophylactic neuroprotection against stroke: low-dose, prolonged treatment with deferoxamine or deferasirox establishes prolonged neuroprotection independent of HIF-1 function. *J. Cereb. Blood Flow Metab.* 31 1412–1423. 10.1038/jcbfm.2010.230 21245873 PMC3130314

[B90] Zhou D. Matchett G. A. Jadhav V. Dach N. Zhang J. H. (2008). The effect of 2-methoxyestradiol, a HIF-1αinhibitor, in global cerebral ischemia in rats. *Neurol. Res.* 30 268–271. 10.1179/016164107x229920 17716391 PMC3563278

[B91] Zhou J. Li J. Rosenbaum D. M. Zhuang J. Poon C. Qin P. (2017). The prolyl 4-hydroxylase inhibitor GSK360A decreases post-stroke brain injury and sensory, motor, and cognitive behavioral deficits. *PLoS One* 12:e0184049. 10.1371/journal.pone.0184049 28880966 PMC5589177

